# Comparing supervised machine learning and large language models in title-abstract screening

**DOI:** 10.1186/s13643-026-03199-6

**Published:** 2026-06-09

**Authors:** Marco F. Aigner, Matthias Ganzinger, Pascal Probst, Moritz Rinckens, Thomas M. Pausch

**Affiliations:** 1 Institute of Medical Informatics, Heidelberg University, Heidelberg, Germany; 2 Department of Surgery, Cantonal Hospital Thurgau, Frauenfeld, Switzerland; 3 EVIglance AG., Hüttlingen, Switzerland; 4 Department of General, Visceral and Transplant Surgery, Heidelberg University Hospital, Heidelberg, Germany

**Keywords:** Systematic review, Title/abstract-screening, Supervised machine learning, Large language model

## Abstract

**Background:**

Systematic reviews require reviewers to decide on the eligibility of large numbers of articles derived from database searches. To accelerate review conduct while continuously more literature gets published, past studies proposed automating the title/abstract-screening step by either supervised machine learning or large language models. Because prior studies mainly compared results within the same model family, we directly compared common TF-IDF-based supervised baselines and a zero-shot, criteria-prompted, and open-weight large language model on the same data to discuss whether, and in which scenarios, they are feasible for review screening automation.

**Methods:**

We predicted the eligibility of labeled articles by four supervised machine learning models (Naïve Bayes, support vector machine, random forest, logistic regression) and one large language model (Llama-3.1—8B-Instruct). Articles were labeled with eligibility as decided by human reviewers in six systematic reviews. We evaluated the performance by binary confusion matrices and calculated recall, specificity, precision, F1-score, and accuracy over a thousand bootstrap samples each. We compared these results to a reported performance of 0.86 (recall) and 0.79 (specificity) in single human reviewers.

**Results:**

Model performance varies greatly between the data sets. Except for Naïve Bayes, recall and specificity are closer aligned in the supervised machine learning models compared to llama. Averaged across all datasets, llama matches human recall and the Naïve Bayes classifier exceeds it, while both fall behind human specificity. Conversely, logistic regression, random forest and support vector machine fall behind human recall while all three exceed human specificity.

**Conclusions:**

Both supervised machine learning and large language models achieve recalls close to or above those of human reviewers. The supervised machine learning models achieve a higher harmonic mean of recall and specificity, while the llama model is more sensitive. Considering the reliance on training data and the all-or-nothing automation with supervised machine learning, this study’s results warrant their use in the extension of pre-existing, non-critical, systematic reviews. Contrarily, as large language models decide on articles individually and as they provide comprehensive, discussable, reasoning they may be used in tandem with human reviewers while the performance of ensembles of large language models is yet to be analyzed.

**Supplementary Information:**

The online version contains supplementary material available at 10.1186/s13643-026-03199-6.

## Background

A systematic review (SR) is frequently used to synthesize research results, for example in medicine, psychology, or ecology. Review synthesis mitigates the biases within single studies and creates robust evidence. Students and researchers use this evidence to overview research areas, to generate hypotheses and to develop high-quality clinical guidelines [1]. Evidence-based practice as well as preclinical research [2] therefore rely on a regular conduct of SRs to provide patients with the best possible care according to the latest evidence.

Conducting a SR is time intensive. From formulating a research question to synthesizing and publishing the findings, the highly structured SR process [3, 4] has been reported to be 61 weeks [5] and 67.3 weeks [6] long on average. However, as new studies change the outcomes of a SR every 5.5 years overall, and 2.7 years in fast-developing medical fields as cardiology [7], SR requires regular updates. In the meantime the number of published studies increases exponentially [8, 9].

Besides looking into dynamic publication formats such as regularly updated living SRs [10], one proposal to keeping SRs up to date with the latest evidence is to accelerate their conduct by automating title/abstract-screening. During this laborious SR process step, reviewers read the titles and abstracts of potentially thousands of articles yielded by a literature database search. They then filter out irrelevant articles by pre-defined eligibility criteria, and eventually include around 3% of all articles [6]. The common practice of employing at least two reviewers [11], as individual reviewers have been found to miss up to 13% of relevant articles [12, 13], effectively doubles the required work.

As deciding on an article’s eligibility is a binary classification task, past research has investigated how supervised machine learning (SML) models automate title/abstract-screening by predicting a class (“include” or “exclude”) from an input (an article’s text) [14]. Challenges with SML included an imbalance between many excluded and few included articles in the training data and assessing how a model performs on new data. A few recent studies [15–17] have evaluated automating title/abstract-screening by large language models (LLM) which decide on the inclusion of an individual article given its text and corresponding eligibility criteria.

Reproducibility is limited as many studies investigate different models and data and report different results without providing access to the raw data. Many of these studies are inconclusive on the feasibility of screening automation and all of them only compare their results to models of the same type [14].

**Fig. 1:**
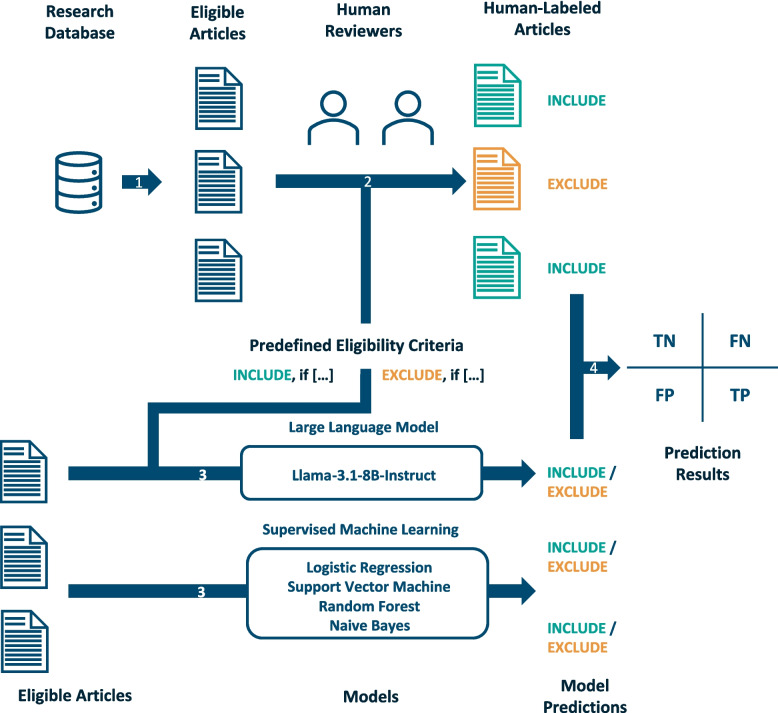
Systematic Review process and study design. Within the systematic review process, initial database searches yield lists of eligible articles for the review (1). Human reviewers classify these articles to either include or exclude using predefined eligibility criteria (2). We trained supervised machine learning models to predict the article’s classes using the labels by human reviewers and prompted a large language model to classify an article based on the article’s title and abstract together with corresponding eligibility criteria (3). We evaluate the models’ performances through confusion matrices and performance scores by comparing the human labels with the predicted classes (4). TN = true negative, FN = false negative, FP = false positive, TP = true positive

In this study we therefore classified six SR datasets using both SML and LLMs (see Fig. 1) guided by the following research questions: (1) how do SML and LLM models perform in classifying articles in direct comparison? (2) Are the selected models ready for practical use in SRs, and if so, in which scenarios? By answering these questions, we aim to contribute towards bringing automation of title/abstract-screening into practice to speed up the conduct of SRs. To establish a first baseline comparison, we decided to focus on Term Frequency–Inverse Document Frequency (TF-IDF)-based approaches for SML with standard classifiers and a zero-shot approach with an open-weight LLM.

## Methods

### Study design

We conceptualized a comparative evaluation study to assess the performance of both SML and LLM in title/abstract-screening against retrospectively collected, human-labeled “gold standard” datasets from real SR.

### Datasets

#### Sources

Out of the six datasets we used, five are open source. We accessed these open-source datasets from the SYNERGY [18] dataset on study selection in SRs. Using the identifiers within SYNERGY, we retrieved titles and abstracts from the OpenAlex [19] index through the PyAlex [20] library for each article. The data on pancreatic surgery was directly provided by P.P. and M.R.

#### Analysis

We processed the data in Python using Pandas [21] and Polars [22] dataframes. First, we calculated the distribution of class labels (i.e., how many articles were included or excluded respectively). We searched for noise (i.e., tokens that do not belong to the original article) by manually inspecting the articles’ texts. We automatically labeled the text languages using the fast-langdetect [23] library. After manually correcting the labels of articles that were incorrectly flagged as non-English, we used this column to inspect the number of non-English articles per dataset.

We searched each dataset for duplicates using the *polars.DataFrame.filter* function with an article’s *title* as well as identifiers such as *doi, pubmed_id, openalex_id, webofscience_id*, and *central_id* as parameters.

We calculated the number of words in the titles and abstracts respectively, by tokenizing them using the *word_tokenize* function of the natural language toolkit [24] and counting the number of tokens. We then inspected the distributions of the number of words per dataset. We compared the number of articles without titles or abstracts before and after our preprocessing. Lastly, we generated word clouds to visually help determine whether the vocabulary within the texts accurately reflects the topics of the datasets.

#### Preprocessing

We preprocessed the data stepwise and saved their state after each step in respective files which we share together with this manuscript. We mapped the different class labels to a Boolean column called “include” by associating a *label_included* value of 0 (SYNERGY) and a *state* value of 3 (pancreatic surgery) with a value of “False” and all other values with “True”. We retrieved the title and abstract of each article within SYNERGY through the PyAlex [20] library. For each article with a PubMed ID, we retrieved additional metadata from the NCBI Entrez Programming Utilities (E-utilities) interface [25] using the Metapub library [26]. If an article was missing either title or abstract in the original dataset, we also retrieved those from E-utilities, if possible. We removed whole articles from the datasets if they were duplicates, had non-English or exceptionally long abstracts, or no abstract at all. We considered an abstract exceptionally long if it exceeded 489 words, which was the 95% quantile across all datasets. We further removed HTML, digits and special characters from the texts using the *beautifulsoup * [27] library and regular expressions.

### Models

#### Supervised machine learning

We used four types of classifiers on the SML side: Naïve Bayes, logistic regression, random forest, and support vector machine. We chose these four classifiers as they are functionally diverse, well understood and as they have already been used in related literature [28–35]. The model implementations (*ComplementNB, LogisticRegression, RandomForestClassifier, SVC*) were taken from the scikit-learn [36] library. We set the *class_weight* parameter to "*balanced*" for all models except Naïve Bayes, in which *class_weight* does not exist, to control the heavy imbalance between excludes and includes. Otherwise, we used models with their default parameters.

#### Large language model

The large language model used in this study was Llama3.1—8B-Instruct [37]. We chose Llama3.1—8B-Instruct because, out of the models that had been used in related studies [38–40], it was the latest model from the Meta Llama family of models which distinguishes itself by its relatively open-weight nature and its ability to infer locally. We accessed the model through *AutoClasses* [41] within the Hugging Face [42] platform. After downloading the model weights from Hugging Face, we used *AutoTokenizer.from_pretrained()* with *padding_side* set to “*left*” and *tokenizer.pad_token_id* set to “*tokenizer.eos_token_id*”, *AutoConfig.from_pretrained()*, and *AutoModelForCausalLM* with 16-bit precision and *pad_token_id* set to *tokenizer.eos_token_id*. The *do_sample* parameter was set to false to minimize output variability. Therefore, the model used greedy decoding, and the temperature parameter did not affect generation.

We report this study with reference to the TRIPOD-LLM guideline [43]; the completed checklist is provided as Supplementary information.

### Classification

We trained the SML models on training subsets of the data and evaluated each model on the remaining test set using the *train_test_split* function from scikit-learn [36] on all datasets. In *train_test_*split we set a *test_size* of 0.3, a *random_state* of 42 and stratified by the class (*y*) to account for class imbalance.

#### Supervised machine learning

The SML classification was written in Jupyter notebooks and run locally on a consumer-grade laptop computer (AMD Ryzen 7 6800U CPU, 16 gigabyte RAM). SML-specific preprocessing included lowercasing, reducing the words to their lexical forms (lemmas) using the *WordNetLemmatizer* class of the natural language toolkit [24] optimized by positional tags, and removing English stopwords from the natural language toolkit list. To control for class imbalance, we randomly removed excluded articles using the scikit-optimize [44] library, which integrates with scikit-learn. Our pipeline consisted of a *TfidfVectorizer* (scikit-learn), followed by a *RandomUnderSampler* (scikit-optimize) with *sampling_strategy* set to “auto” and *random_state* set to “42” followed by the respective model. Running this pipeline on each combination of model and dataset, we fitted the models on the training data and let them predict the test data afterwards.

#### Large language model

As the LLM classification relied on eligibility criteria to decide on an article’s inclusion, we manually extracted those from the underlying SRs (Supplementary data Table S2). We imported the eligibility criteria from.txt-files and saved them into two variables: one for inclusions and one for exclusions. Thus, the criteria were incorporated into the prompt as structured free-text inclusion and exclusion fields, rather than as a formally encoded schema. We wrote the LLM classification pipeline within a Python script which we ran on the bwHelix high performance computing cluster located at Heidelberg University. Although the model can run on computers with consumer grade graphics processing units, we chose to use bwHelix to speed up the classification in context of the extraordinarily high number of articles especially within the pancreatic surgery dataset. We used a HuggingFace *pipeline* [45] with *task* set to “text-generation”, 16-bit floating point precision and a maximum number of new tokens of 150. We asked the model to decide on an article’s inclusion or exclusion given its title, abstract, and eligibility criteria using a modified version of the prompt from Guo et al. [15] (Extended data Fig. S1). We extended the prompt by asking the model to explain its decision. We let the model classify the datasets in batches of 16 articles at a time and saved its decision together with its reasoning, title, DOI, and PubMed ID. Because of its size, we split the pancreatic surgery dataset into six parts, which we classified in parallel and combined again later.

### Evaluation

We evaluated all models on the same test subsets of each dataset. As such, we are aiming for a single-split baseline benchmarking of the models.

#### Confusion matrix

First, we calculated the absolute and normalized confusion matrices by providing lists of the predicted and true inclusions to scikit-learn’s *confusion_matrix* function. From each model’s confusion matrix, we obtained the number of true negatives (TN), false positives (FP), false negatives (FN), and true positives (TP). These values allowed us to calculate additional performance measures.

#### Performance measures

In title/abstract-screening, recall $\left(1\right)$ [0,1] describes the number of articles that were correctly labeled as included. In this context, optimizing for recall means aiming to find all relevant literature at the risk of also including false positives. Recall is the normalized number of true positives and is therefore directly present in the bottom right cell of a normalized confusion matrix.

1 $$\begin{array}{c}Recall=Sensitivity= True\;Positive\;Rate= \frac{TP}{{TP} + {FN}}\end{array}$$

Specificity $\left(2\right)$ [0,1] is the proportion of correctly classified negatives amongst all negatives. As proven by Kusa et al. [46], specificity is equal to the normalized version of the work saved over random sampling (nWSS) metric, which is commonly used in studies on automating title/abstract screening [47]. Note that specificity is the normalized number of true negatives and therefore present in the upper left cell of a normalized confusion matrix.

2 $$\begin{array}{c}Specificity=True\;Negative\;Rate=nWSS= \frac{TN}{{TN} + {FN}}\end{array}$$

Precision $\left(3\right)$ [0,1] is the proportion of correctly identified articles that were included among all articles that were included.

3 $$\begin{array}{c}Precision=Positive\;Predictive\;Value= \frac{TP}{{TP} + {FP}}\end{array}$$

The ${F}_{\beta }$ measure [0,1] combines precision and recall $\left(4\right)$. Here, the parameter β weights the influence of both values with β > 1 emphasizing recall over precision and β < 1 emphasizing precision over recall. Studies on classification tasks often report the ${F}_{1}$ measure, which is the harmonic mean of precision and recall. The ${F}_{\beta }$ measure can also be described by the elements of a confusion matrix $\left(5\right)$. Note how the second version of the formula $\left(5\right)$ reveals how ${F}_{\beta }$ ignores the number of true negative predictions.

4 $$\begin{array}{c}{F}_{\beta }=\left(1+{\beta }^{2}\right)*\frac{{Precision}*{Recall}}{\left({\beta }^{2}* {Precision}\right)+{Recall}}\end{array}$$

5 $$\begin{array}{c}{F}_{\beta }=\frac{\left(1+{\beta }^{2}\right)*{TP}}{\left(1+{\beta }^{2}\right)*{TP}+{\beta }^{2}*{FN}+{FP}}\end{array}$$

Accuracy $\left(6\right)$ [0,1] is defined as the proportion of correct classifications compared to the whole. Within the context of highly imbalanced SR datasets, in which a poorly fitted SML model might exclude most articles, the informational content of accuracy is limited.

6 $$\begin{array}{c}Accuracy= \frac{{TP}+{TN}}{N}\end{array}$$

#### Confidence intervals

We report performance measures together with confidence intervals for each combination of dataset and model. Apart from the F1-score, we calculated confidence intervals for every measure using bootstrap-sampling. We calculated the F1-score using the deterministic delta-method proposed by Takahashi et al. [48], as implemented in the *confidenceinterval* python library [49], to account for the fact that F1 is being calculated by two correlated measures (precision and recall) from a binomial distribution. To calculate confidence intervals for all other performance measures, we bootstrap-sampled (sampling with replacement) the predictions of each estimator on each dataset a thousand times. We calculated the performance measures for each of the 1000 bootstrap samples, which resulted in a distribution of measures. We took the mean average of this distribution as the point measure and derived the lower and upper bounds for a 95% confidence interval by calculating the 2.5th and 97.5th percentile of the distribution each. We rounded the mean as well as the lower and upper bounds of the confidence interval to two decimal places.

## Results

### Datasets

#### Sources

All six data sets used in this study are based on published SRs. These SRs cover a variety of medical topics from *pancreatic surgery* [50] and *animal depression* [51] to drug class reviews related to *attention deficit hyperactivity disorder* [52] (ADHD), *atypical antipsychotics* [53], *calcium channel blockers* [54], and *oral hypoglycemic* [55] (see Table 1). The proprietary pancreatic surgery dataset stems from a living SR by the International Study Group on Pancreatic Surgery (ISGPS) [50] which is available online as the evidence map of pancreatic surgery (www.EVIglance.com). All other datasets are from the open-source repository *SYNERGY* and have been used in studies [17, 29, 47, 56, 57] that investigate automation of title/abstract-screening.

**Table 1:** Overview of datasets

Dataset	*n*	Removed by data cleaning	Test set	Test include
ADHD	851	6.2%	240	2.5%
Atypical antipsychotics	1120	6.3%	9076	5.6%
Calcium channel blockers	1218	7.3%	339	8.3%
Oral hypoglycemics	503	8.9%	315	13.3%
Pancreatic surgery	34,180	11.5%	508	14.6%
Animal depression	1989	15.0%	138	26.0%

The datasets consist of between 851 and 34,180 articles. The removal of duplicates, non-English articles, and such with exceptionally long abstracts removed between 6 and 15% of articles from the datasets. The test datasets are one-third of the size and have the same rate of included articles as the whole datasets do

#### Analysis

Each data set contains the title, abstract, and class label of its articles. Within the *SYNERGY* datasets, inclusion during title/abstract-screening is binary encoded, while in the pancreatic surgery data, the inclusion is encoded as one of many possible study states. Other data fields may include a study type, a literature id referring to PubMed, Cochrane Central, Web of Science, or OpenAlex, and the article’s first author. Each dataset further holds a digital object identifier.

**Fig. 2:**
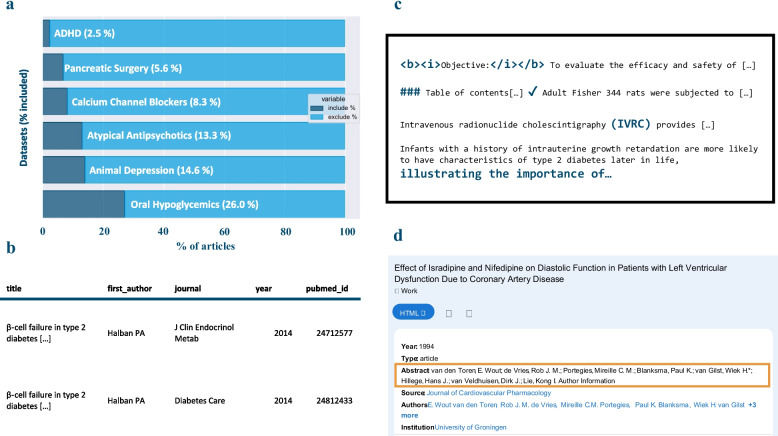
Findings from the dataset analysis. **a** The relative proportion of included articles ranges from 2.5 to 26.0% **b** Example of a duplicate. The two instances are the same article, which has been published twice and therefore received two different pubmed ids. If the id of either article would be missing, the duplicate could not be automatically detected. **c** Examples of noise within the datasets that do not contribute information include HTML- and Markup tags, inconsistently used abbreviations and abruptly ending, incomplete texts. **d** The OpenAlex entry of an article from the calcium channels dataset. A list of authors is wrongfully in place of the abstract

The ratio of included articles in the data sets ranges from 2.5 to 26.0% with a median of 10.8% across all datasets (Fig. 2a).

Examples of noise within the abstract texts are HTML and Markdown elements, special characters, abbreviations, or incomplete (i.e., truncated) abstracts. In some instances, a list of authors is wrongfully present in the abstract field (see Fig. 2d).

Non-English titles are present in 578 articles (1.45% of all articles), and non-English abstracts are present in 35 articles (0.09% of all articles).

Across all datasets there are 530 cases in which two articles from the same dataset have an identical attribute, such as title, or an identifier (Fig. 2b, Supplementary data Table S1).

The median title length is 13.5 words, and the median abstract length is 240.0 words. Outliers range from one word to 88 words for titles, and zero words to 78,903 words for abstracts. Very short abstracts are often cut off and end abruptly while very long abstracts include non-English versions of the same abstract or completely unrelated text.

The most frequently occurring words within a dataset are closely related to its topic as visualized by the word clouds (Supplementary data Fig. S1).

#### Preprocessing

Additional metadata were added to the datasets using the PubMed E-Utilities [25, 58] API. This included information such as authors, bibliographic details (publication year, journal, digital object identifier), PubMed-related information (PubMed type, publication types, medical subject headings) and identifiers relating to other repositories (Web of Science, Cochrane Central, OpenAlex). The API call further retrieves the missing titles for all datasets except animal depression. While the number of missing abstracts decreases, 5.76% of articles remain without an abstract on average (minimum 2.47%, maximum 12.84%).

Data cleaning has removed a median of 80 articles from each dataset which corresponds to 8.1% of the original dataset sizes (Supplementary data Fig. S2).

### Classification

On average, the SML models take 40 s for training and predicting. They produce a.csv file with the true labels and the predictions side by side. The.csv output of the Llama pipeline additionally contains a column with the model’s justification and takes between three and 34 min to predict, depending on the dataset.

**Fig. 3:**
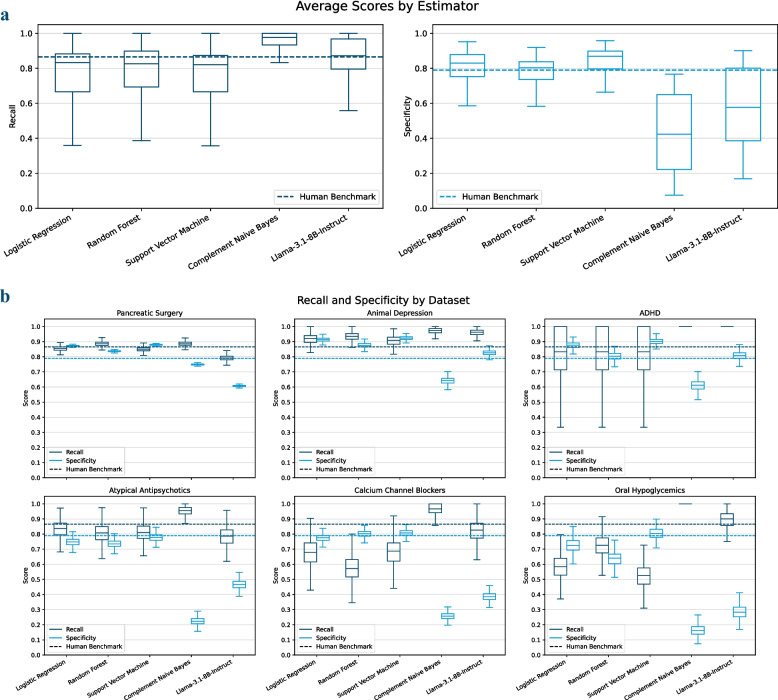
Estimator scores on average and per dataset. **a** Recall and specificity averaged across all datasets. In comparison with a human benchmark, all models achieve similar recall, and logistic regression, random forest, and support vector machine achieve similar specificity. Narrower confidence intervals indicate how consistent a model performed. **b** Recall and specificity by dataset. Scores are higher in the pancreatic surgery, animal depression, and ADHD dataset compared to the other three. With supervised machine learning, recall and specificity tend to be closer together than with Llama, which consistently achieves higher recalls, even on the lower performing datasets. The boxplots have been produced by the matplotlib.axes.Axes.boxplot function in python on 1000 bootstrap samples of model predictions per dataset. The box extends from the first quartile (Q1) to the third quartile (Q3) with the center line representing the median. The whiskers extend from the box to the farthest data point lying within the 1.5 × the inter-quartile range (IQR) between Q1 and Q3. Outliers have been present but omitted from the figure for clarity

All classification results are based on the test datasets (30% of full datasets, stratified for inclusion rates, Table S1) Confusion matrices were prepared for each model to visualize the results (see Extended data Fig. S2 and Supplementary data Fig. S3 to S7). Additionally, performance scores with 95% confidence intervals were calculated for each combination of dataset and model (see Fig. 3, Extended data Table S1, and Supplementary data Fig. S8 and S9).

The model performances vary between the data sets in different ways (Fig. 3b). Models have achieved human performance, meaning that they have met or exceeded both a recall of 0.865 and a specificity of 0.79 on the three datasets *animal depression* (all except for the Naïve Bayes classifier), *pancreatic surgery* (logistic regression, random forest, support vector machine), and *ADHD* (llama). The models have achieved lower scores on the drug report datasets and could neither achieve human level recall nor specificity on *atypical antipsychotics* (all except for Naïve Bayes), *calcium channel blockers* (llama), and *oral hypoglycemics* (logistic regression, random forest). Except for llama on *ADHD* and *oral hypoglycemics*, and Naïve Bayes on every dataset, no model achieved human-level recall without considering the specificity.

Model scores are affected differently by varying performance. With logistic regression, random forest and support vector machine classifiers, recall and specificity are relatively close to each other and both relatively lower in the drug report datasets compared to *pancreatic surgery* or *animal depression*. Contrary, on the same drug report datasets the Naïve Bayes and Llama models achieve recall rates comparable to human performance, while achieving much lower specificity.

Across all datasets, the models achieve a recall close to (logistic regression, random forest, support vector machine), or above (Naïve Bayes, Llama) the performance of one human reviewer. Specificity is the other way round, as logistic regression, random forest, and support vector machine score above, while Naïve Bayes and Llama score below the performance of one human reviewer. By the size of the error-bars on the average metrics (Fig. 3a), Naïve Bayes and Llama are more consistent than the other models in achieving high recalls, while it is the other way around with specificity.

## Discussion

In this study we used four SML classifiers and the Llama-3.1—8B-Instruct LLM to predict the eligibility of articles from the six same datasets on SRs to compare their performance in title/abstract-screening directly to each other. To increase comparability, we refined the models as little as possible and report on the same performance measures. Although model performance varies greatly between datasets, both the SML models and the LLM achieve recall values close to or above those of human reviewers. SML achieves higher F1-scores than the LLM, while the latter is more sensitive towards true positives. Based on our results we discuss the scenarios in which the selected models could be used for automation in practice. Our comparison should be interpreted narrowly as a benchmark between classical TF-IDF–based supervised baselines trained on review-specific labels and a zero-shot, criteria-prompted open-weight LLM, rather than as a definitive comparison between all possible SML and LLM approaches. 

### Limitations

The results of this study are only partially comparable to those of other studies, as many of these vary in terms of datasets, models and other methods that they used. We intentionally chose datasets with varying class imbalances compared to the 3–6% common in health-related SRs [59] to see whether different inclusion rates would influence classification performance.

Our intention was to establish a baseline benchmark for SML and LLM screening, not to optimize a specific model. For instance, a single standard zero-shot prompt was employed for the LLM evaluation. Further research could include more elaborate prompting approaches, such as few-shot examples or in-context learning.

#### Large language models

In contrast to SML, LLM have some properties that cannot easily be controlled by their users. For instance, the text corpus on which the Llama models were trained is not publicly available or even documented. Thus, the datasets of this study as well as SR based on them might already be part of the training corpus. Therefore, the LLM might be overfitted to retrospective data.

#### Dataset preparation

Without access to the original data, errors, such as a list of authors instead of the abstract found within OpenAlex, carry over from the alternative source to the study data.

Due to the size of the datasets, we relied on automation (filtering non-English abstracts, duplicates) and heuristics (removing lengthy abstracts) to search for noise in the data. It is possible that there are still duplicates in the pancreatic surgery dataset, as we did not manually cross-check 490 articles with matching titles. Such duplicates would receive greater weight during SML training and in the evaluation.

#### Classification

A limitation inherent to title/abstract-screening itself is that the labels used to train and evaluate the models were assigned by human reviewers, who are susceptible to subjectivity and interpretation. For example, from two reviewers who are not sure whether a given article matches the eligibility criteria, one might include it during title/abstract-screening, effectively delaying the decision, while the other one might already exclude it. Note how this way, model performance is evaluated relative to prior human labels instead of an objective truth, which introduces a variance/an error to the benchmark which automation studies evaluate against.

To respect the individual strengths and weaknesses of the models, and to set a benchmark for future investigations, we did not refine the models. Besides random undersampling and balanced class weights to handle class imbalance, we did not set any SML hyperparameters.

Possible optimizations for SML include vectorization methods other than TF-IDF (see Methods), such as Word2Vec [60] and GloVe [61], other sampling strategies such as no sampling, oversampling, other forms of undersampling, or synthetic minority oversampling technique (CDSMOTE) [62] through systematic optimization approaches such as grid-search, random-search, or optimization based on sequential models [44].

Possible optimizations for LLM include a systematic comparison of models with different numbers of parameters, varying prompts and refining the formulation of eligibility criteria, possibly with the help of an LLM. We did not perform formal prompt-sensitivity analyses because the study aimed to benchmark a single fixed zero-shot prompt template rather than optimize prompting performance. Because the LLM was pretrained on large web-scale corpora, we cannot exclude that some evaluated abstracts, related review texts, or closely similar examples were present in its pretraining data. Therefore, LLM performance should be interpreted as performance on publicly available retrospective datasets rather than as evidence of guaranteed independence from prior exposure.

### Classification results

When interpreting the results, we prioritize recall over other metrics, because overlooking relevant studies might change the outcome of a SR [8]. Our second most important metric is specificity, which is equivalent to the amount of work saved in subsequent SR steps [46].

#### Performance between datasets

The performance of the models varies between the datasets and the highest scores have been achieved on the datasets on pancreatic surgery and animal depression. This discrepancy suggests systematic differences between the datasets. The origin of these discrepancies needs systematic analysis, but possible explanations could include attributes such as class imbalance or the total number of articles in SML, or the formulation of eligibility criteria in the LLM. Further, errors such as duplicate articles or corrupted texts cannot be ruled out at this point. We agree with Moreno-García et al. [29] that the data quality could be higher in more recent datasets as they are from domains in which authors tended to write more systematically, which could explain the correlation between a dataset’s recency and the performance the models achieve on it.

We expect that removing lengthy abstracts had little influence on the classification results, as datasets with longer abstracts (atypical antipsychotics and calcium channel blockers) perform similarly to those with shorter abstracts (oral hypoglycemics). Further, we see no clear pattern between inclusion rates and classification performance. Underperforming datasets had inclusion rates that were both very low (calcium channel blockers, 8.3%) and very high (oral hypoglycemics, 26.0%), while the best performing dataset on animal depression was in between (14.0%).

#### Performance comparison between SML and LLM

Although the SML models surpass the LLM by absolute performance measures, this difference is often of unclear significance as the confidence intervals overlap.

The models’ different performance across the datasets might be explained by how they function. SML models learn patterns between term frequencies and class labels. In this study, well-fitted models, such as logistic regression, random forest, and support vector machine, have similar recall and specificity, both of which decrease in lower-performing datasets. The Naïve Bayes classifier is an example of a poor fit, as it achieves high recalls by including most of the articles and neglecting the “exclude” class. The LLM achieves high recall and lower specificity but is not influenced by performance measures; rather it is influenced by contextual word embeddings and inclusion criteria alone. While an LLM has no semantic understanding like humans, its behavior seems to mimic the human behavior of including articles when in doubt.

#### Comparison with other studies

Comparing this study’s results with a study that applied Llama-3–70B-Instruct on the animal depression dataset [17], the two Llama models performed similarly well in title/abstract-screening, as indicated by point estimates and confidence intervals. This finding contrasts with official benchmarks, which show that Llama-3–70B-Instruct outperforms the 8B version [37]. These results raise the question of whether, for this specific use case, smaller models with fewer parameters suffice, which could allow more users to use them locally.

Two other studies [15, 16] which evaluated GPT-4 criticize the model’s low recall. It is unclear whether this is due to the model or other factors since only one study applied the same prompt as this study, but both used different datasets.

A study by Moreno-García et al. [29] combined support vector machine and random forest models with a BART [63] model to classify articles based on a single label such as “animal depression”. While this hybrid approach achieves similar performance to this study, relying on a single label seems unintuitive when dealing with finely nuanced inclusion criteria in a set of search results that already holds similar articles.

Consistent with our findings, other studies report the best performance for the support vector machine [64] model, together with undersampling and unigram vectorization [65, 66]. While training on datasets enriched with unrelated articles increased classification performance in two studies [57, 67], this could potentially originate from models learning to distinguish whole topics, which would make them miss fine nuances in the eligibility criteria. Their finding that incorporating medical subject headings (MeSH) [68] in the training data led to better results seems logical, since MeSH is a controlled vocabulary, designed to systematically describe factors such as subjects, methods, and types of articles.

### Application in practice

#### Accessibility

To us, an accessible model is one that is freely available and easy to use. Both SML models and the Llama-3.1—8B-Instruct model are open-source, free of charge, and run on consumer-grade hardware.

However, training and evaluating SML models requires dedicated experience in programming languages and machine learning theory. While integrating an LLM into an automated workflow also requires dedicated knowledge, its dialogue-like interface and output in natural language increase its comprehensiveness, making it more accessible in our eyes.

#### Trustworthiness

We consider a model to be trustworthy if it is reliable and comprehensive.

Reliability, as defined by consistent performance under the same conditions, has yet to be achieved, given the heterogeneity of past studies, and will require future replication studies that adhere to the same methods.

Regarding comprehensiveness, SML models are generally difficult-to-understand “black boxes”, whose performance on new data can only be estimated using test sets. On the other hand, the reasoning which an LLM provides next to the decision on each article’s inclusion, if prompted for, enables human reviewers to double-check the output, similarly to how two human reviewers resolve conflicting decisions.

As people have been experimentally shown to be less likely to automate tasks within the medical domain compared to other domains, or if human wellbeing was at risk due to the automation [69], we expect the increased comprehensiveness of the LLM over SML to contribute to user acceptance in a context where missing relevant studies can alter patient treatment.

#### Practical implications

Living SRs that provide past screening decisions to train on seem most suitable for automating by SML. However, screening by SML is ineffective if the scope of the review changes over time, as past training data cannot account for this. Since LLMs do not require training data, they can be used for both living and new SRs.

In our study, the performance of both SML and LLM is sufficient for fully automating uncritical scoping reviews. In critical scenarios, like meta-analyses, where the exclusion of relevant studies might alter medical practice, LLMs could serve as one out of two reviewers, reducing the workload in a dual-reviewer setting by half.

Active learning frameworks, such as ASReview [70], work by training an SML model on sorting by relevant articles. Active learning begins with a calibration by manually labelling a few examples of included and excluded articles. Each subsequent decision on article inclusion iteratively re-trains a model on sorting by the most likely included articles. With likely inclusions sorted first, the human reviewer remains in control of the decision while achieving higher recalls faster compared to random sampling. Integrating an LLM into active learning could combine the benefits of both approaches. In this case, both a human reviewer and an LLM would screen articles in an active learning environment and then resolve conflicts regarding different decisions.

We argue that automated processes must be comprehensive and verifiable until they can match the performance of teams of human reviewers. This requirement necessitates human’s involvement in the process, and prioritizes saving time over full automation.

The large number of articles that need to be screened during the title/abstract-screening stage makes it impractical to manually copy and paste each prompt together with eligibility criteria and article text into standard chat-like LLM interfaces. Instead, we recommend reviewers to infer LLMs programmatically. Commercial providers often offer paid application programming interfaces to programmatically infer their models. Alternatively, reviewers with access to a graphics processing unit can adapt our code to run LLMs locally using libraries such as Hugging Face [42]. For reviewers who want to use active learning, we recommend ASReview Lab, an all-in-one framework that includes models and tutorials [71]. Deployment decisions should be guided by predefined, recall-oriented, error-control targets. Threshold selection and periodic human auditing should be tailored to the criticality of the review question.

## Future research

Future research should focus on curating datasets, such as SYNERGY, for reuse in studies on the automation of title/abstract-screening. A follow-up study could use this study’s results to investigate how attributes in the datasets and inclusion criteria contribute to classification performance. The dataset on animal depression is especially relevant in this context because all models in this study and in other studies have achieved classification performance comparable to that of expert human reviewers using this dataset. Especially for living SRs, future studies should train on earlier update cycles and evaluate on later waves in order to test temporal generalization under realistic prospective conditions.

## Conclusions

Evidence-based clinical practice as well as preclinical research rely on SRs to provide patients with the best possible care according to the latest evidence. The traditional SR process is laborious, error-prone, and, with new studies constantly being published, not sustainable.

Living SRs that are continuously updated are currently being established and save time compared to traditional publishing in the form of scientific articles. Our results will empower systems like EVIglance to speed up the ongoing screening process.

In practice, a model must be accessible, performant, and trustworthy to be used in title/abstract-screening. While both types of models are similarly accessible as they can run on consumer-grade hardware, we consider Llama-3.1—8B-Instruct to be more trustworthy, as it explains its inclusions in natural language which enables human reviewers to validate their correctness. This attribute could potentially allow for teams in which LLMs and human reviewers collaborate in title/abstract-screening. SML models, on the other hand, are incomprehensible black boxes which are not trivial to implement and interpret for reviewers with only a medical background.

These conclusions are limited to the presented benchmark of classical TF-IDF–based supervised baselines and a zero-shot open-weight LLM, both evaluated with minimal parameter tuning.

## Supplementary Information

Supplementary Material 1: Extended data Figure S1: The prompt template provided to Llama-3.1—8B-Instruct. We adapted the prompt from Guo et al. [15] in two ways. First, we additionally instructed the model to provide reasoning for its decision. Second, we separated the instructions into a system- and a user-prompt. The system prompt instructs the model to act as a researcher and to use provided criteria to decide whether to exclude or include an article. The user prompt provides title, abstract, inclusion, and exclusion criteria. Extended data Figure S2: Classification results for each combination of model and dataset There is one normalized confusion matrix for each combination of model and dataset each. Extended data Table S1: Performance measures with 95% confidence interval for each combination of dataset and model. All models have been evaluated on the test subsets. Point estimates and 95% confidence intervals have been calculated by taking the mean, the 2.5th and the 97.5th percentile of the scores that have been calculated over 1000 bootstrap samples. For each dataset, the highest score is printed in bold and in cyan color. Supplementary data Figure S1: Word Count Analysis More frequently appearing terms are printed larger within the word clouds. After filtering out common stopwords, the most frequent terms are characteristic to the topics of the datasets. Supplementary data Figure S2: Reduction in articles without titles or abstracts The bars show the relative amount of articles with either missing titles or abstracts before (dashed) and after (solid) calling the PubMed Entrez interface. The number of articles without titles has been largely reduced, and the number of articles without an abstract has been reduced to a varying degree. Supplementary data Table S1: Number of duplicate articles by attribute and dataset. All datasets contain at least two articles with an identical title. The datasets on animal depression and pancreatic surgery further contain articles with duplicate identifiers. Supplementary data Table S2: Inclusion criteria provided to Llama-3.1—8B-Instruct. We manually extracted the inclusion criteria for the datasets as we manually extracted them from their underlying systematic reviews. During inference, we loaded the criteria into one string for inclusion and exclusion each, and pasted them into the prompt template in Extended Data Fig. 1. Supplementary data Figure S3: Classification results of the logistic regression classifier. Supplementary data Figure S4: Classification results of the random forest classifier. Supplementary data Figure S5: Classification results of the Support Vector Machine Classifier. Supplementary data Figure S6: Classification results of the naïve Bayes classifier. Supplementary data Figure S7: Classification results of the Llama-3.1—8B-Instruct Model. Supplementary data Figure S8: Performance measures for each model grouped by dataset Each subplot represents the combination of one performance metric and dataset. Within each subplot, there is one boxplot per model, showing the score, it has achieved. Supplementary data Figure S9: Performance measures for each dataset grouped by model Each subplot represents the combination of one performance metric and model. Within each subplot, there is one boxplot per dataset, showing the score, it has achieved.

## Data Availability

Except for the proprietary dataset on pancreatic surgery, which belongs to EVIglance Inc, all data are freely available online. The initial datasets containing OpenAlex ids were taken from https://github.com/asreview/synergy-dataset. Copies of the datasets at each step of the study can be retrieved from the project pages on the open science framework 10.17605/OSF.IO/QZB2R and on GitHub https://github.com/MarcoAigner/automated_title_abstract_screening. The GitHub repository contains all the code used in this study (Python scripts and Jupyter notebooks), including preprocessing, analysis, classification, and evaluation.
