# Supplementary material for: Comparing supervised machine learning and large language models in title-abstract screening
Source: Syst Rev. 2026 Jun 9;15:190. doi: 10.1186/s13643-026-03199-6 (PMC13248334; doi:10.1186/s13643-026-03199-6)
Supplement: Supplementary file 1 — Supplementary Material 1: Extended data Figure S1: The prompt template provided to Llama-3.1—8B-Instruct. We adapted the prompt from Guo et al. [15] in two ways. First, we additionally instructed the model to provide reasoning for its decision. Second, we separated the instructions into a system- and a user-prompt. The system prompt instructs the model to act as a researcher and to use provided criteria to decide whether to exclude or include an article. The user prompt provides title, abstract, inclusion, and exclusion criteria. Extended data Figure S2: Classification results for each combination of model and dataset There is one normalized confusion matrix for each combination of model and dataset each. Extended data Table S1: Performance measures with 95% confidence interval for each combination of dataset and model. All models have been evaluated on the test subsets. Point estimates and 95% confidence intervals have been calculated by taking the mean, the 2.5th and the 97.5th percentile of the scores that have been calculated over 1000 bootstrap samples. For each dataset, the highest score is printed in bold and in cyan color. Supplementary data Figure S1: Word Count Analysis More frequently appearing terms are printed larger within the word clouds. After filtering out common stopwords, the most frequent terms are characteristic to the topics of the datasets. Supplementary data Figure S2: Reduction in articles without titles or abstracts The bars show the relative amount of articles with either missing titles or abstracts before (dashed) and after (solid) calling the PubMed Entrez interface. The number of articles without titles has been largely reduced, and the number of articles without an abstract has been reduced to a varying degree. Supplementary data Table S1: Number of duplicate articles by attribute and dataset. All datasets contain at least two articles with an identical title. The datasets on animal depression and pancreatic surgery further contain art [file 13643_2026_3199_MOESM1_ESM.docx]

## Extended Data

{"role": "system", "content": "You are a researcher rigorously screening titles and abstracts of scientific papers for inclusion or exclusion in a review paper.

Use the criteria below to inform your decision. If any exclusion criteria are met or not all inclusion criteria are met, exclude the article. If all inclusion criteria are met, include the article.

Type “include” or “exclude” to indicate your decision. **Briefly explain your decision in one short sentence.**

**Use the format<decision>;<explanation>. Do not type anything else."}**,

{"role": "user", "content": f"title: {article[**'title'**]}\n\nabstract: {article[**'abstract'**]}\n\ninclusion: {**INCLUSION**}\n\nexclusion: {**EXCLUSION**}"}

**Extended Data Fig. 1 | The prompt template provided to Llama-3.1-8B-Instruct**. We adapted the prompt from Guo et al.(15) in two ways. First, we additionally instructed the model to provide reasoning for its decision. Second, we separated the instructions into a system- and a user-prompt. The system prompt instructs the model to act as a researcher and to use provided criteria to decide whether to exclude or include an article. The user prompt provides title, abstract, inclusion, and exclusion criteria.


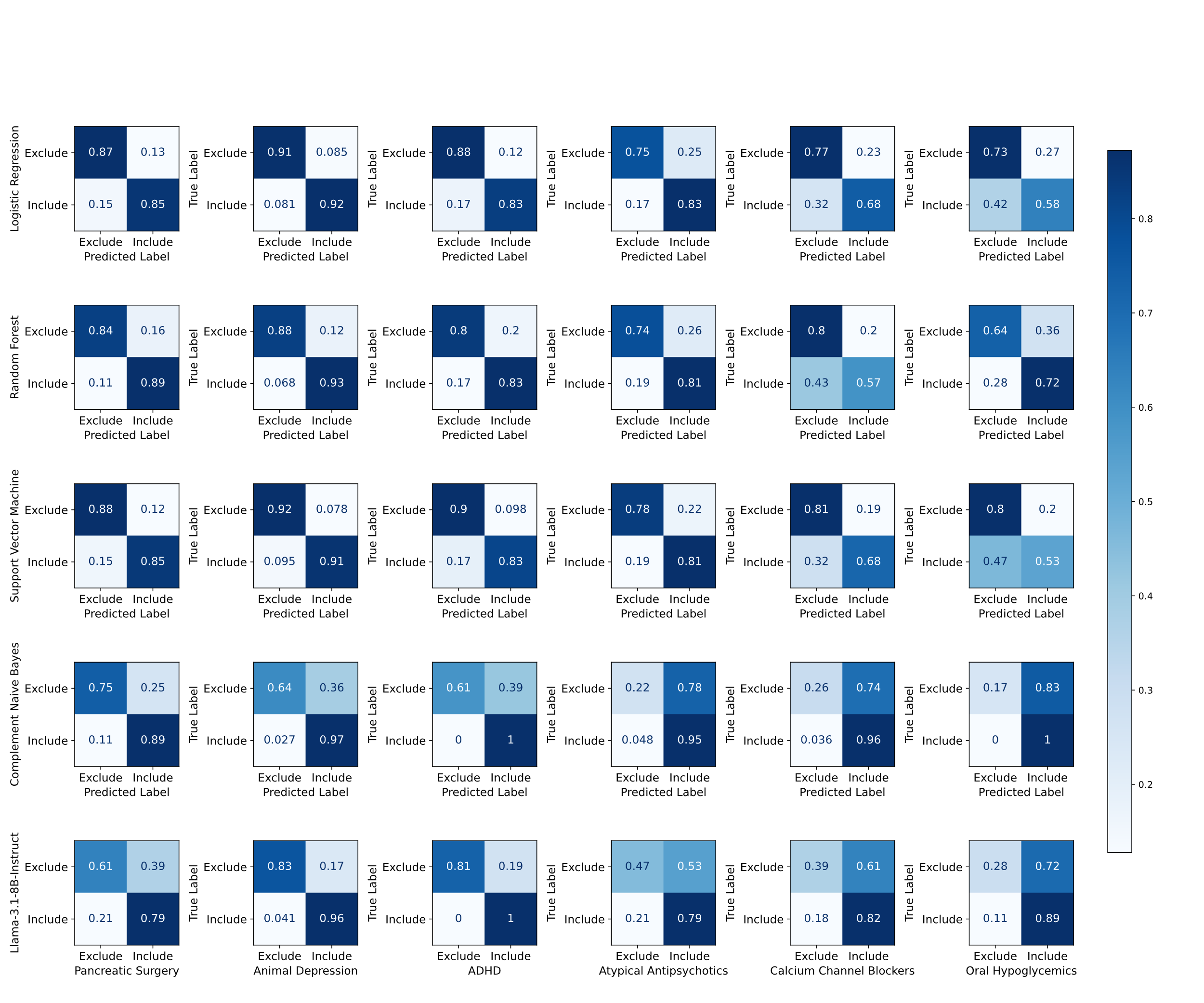


Extended Data Fig. 2 | Classification results for each combination of model and dataset There is one normalized confusion matrix for each combination of model and dataset each.

Extended Data Table 1 | Performance measures with 95% confidence interval for each combination of dataset and model.

| **Dataset** | **Model** | **Recall** | **Specificity** | **Precision** | **F1** | **Accuracy** |
| --- | --- | --- | --- | --- | --- | --- |
| ADHD | Logistic Regression | 0.82 [0.4, 1.0] | 0.88 [0.83, 0.91] | 0.15 [0.03, 0.28] | 0.25 [0.07, 0.43] | 0.87 [0.83, 0.91] |
|  | Random Forest | 0.82 [0.43, 1.0] | 0.80 [0.75, 0.85] | 0.10 [0.02, 0.19] | 0.18 [0.03, 0.32] | 0.80 [0.75, 0.85] |
|  | Support Vector Machine | 0.83 [0.5, 1.0] | **0.90 [0.86, 0.94]** | **0.18 [0.05, 0.33]** | **0.29 [0.09, 0.5]** | **0.90 [0.86, 0.93]** |
|  | Complement Naive Bayes | **1.00 [1.0, 1.0]** | 0.61 [0.54, 0.68] | 0.06 [0.02, 0.11] | 0.12 [0.02, 0.21] | 0.62 [0.55, 0.68] |
|  | Llama-3.1-8B-Instruct | **1.00 [1.0, 1.0]** | 0.81 [0.76, 0.86] | 0.12 [0.04, 0.21] | 0.21 [0.06, 0.36] | 0.81 [0.76, 0.86] |
| Animal Depression | Logistic Regression | 0.92 [0.85, 0.97] | 0.91 [0.89, 0.94] | 0.65 [0.56, 0.74] | 0.76 [0.69, 0.83] | 0.91 [0.89, 0.94] |
|  | Random Forest | 0.93 [0.88, 0.99] | 0.88 [0.84, 0.91] | 0.56 [0.47, 0.64] | 0.70 [0.63, 0.77] | 0.88 [0.86, 0.91] |
|  | Support Vector Machine | 0.91 [0.83, 0.97] | **0.92 [0.9, 0.95]** | **0.67 [0.57, 0.75]** | **0.77 [0.69, 0.84]** | **0.92 [0.89, 0.94]** |
|  | Complement Naive Bayes | **0.97 [0.93, 1.0]** | 0.64 [0.59, 0.69] | 0.32 [0.25, 0.37] | 0.48 [0.41, 0.55] | 0.69 [0.65, 0.73] |
|  | Llama-3.1-8B-Instruct | 0.96 [0.91, 1.0] | 0.83 [0.79, 0.86] | 0.49 [0.4, 0.56] | 0.65 [0.57, 0.72] | 0.85 [0.81, 0.88] |
| Atypical  Antipsychotics | Logistic Regression | 0.83 [0.7, 0.94] | 0.75 [0.7, 0.8] | 0.33 [0.24, 0.42] | 0.48 [0.38, 0.58] | 0.76 [0.71, 0.8] |
|  | Random Forest | 0.81 [0.68, 0.92] | 0.74 [0.68, 0.79] | 0.32 [0.23, 0.4] | 0.46 [0.36, 0.56] | 0.75 [0.7, 0.79] |
|  | Support Vector Machine | 0.81 [0.69, 0.92] | **0.78 [0.73, 0.83]** | **0.36 [0.26, 0.45]** | **0.50 [0.39, 0.6]** | **0.78 [0.74, 0.83]** |
|  | Complement Naive Bayes | **0.95 [0.88, 1.0]** | 0.22 [0.18, 0.27] | 0.16 [0.11, 0.21] | 0.27 [0.2, 0.35] | 0.32 [0.27, 0.37] |
|  | Llama-3.1-8B-Instruct | 0.78 [0.66, 0.9] | 0.47 [0.41, 0.52] | 0.18 [0.13, 0.24] | 0.3 [0.21, 0.38] | 0.51 [0.45, 0.56] |
| Calcium Channel Blockers | Logistic Regression | 0.67 [0.48, 0.84] | 0.77 [0.72, 0.82] | 0.21 [0.13, 0.3] | 0.32 [0.21, 0.44] | 0.77 [0.72, 0.81] |
|  | Random Forest | 0.57 [0.4, 0.75] | 0.80 [0.75, 0.84] | 0.21 [0.12, 0.29] | 0.30 [0.19, 0.42] | 0.78 [0.73, 0.82] |
|  | Support Vector Machine | 0.68 [0.5, 0.84] | **0.81 [0.76, 0.85]** | **0.24 [0.15, 0.34]** | **0.36 [0.24, 0.47]** | **0.80 [0.75, 0.83]** |
|  | Complement Naive Bayes | **0.96 [0.88, 1.0]** | 0.26 [0.21, 0.31] | 0.10 [0.07, 0.14] | 0.19 [0.12, 0.26] | 0.32 [0.26, 0.37] |
|  | Llama-3.1-8B-Instruct | 0.82 [0.67, 0.95] | 0.39 [0.33, 0.45] | 0.11 [0.07, 0.15] | 0.19 [0.11, 0.27] | 0.42 [0.37, 0.48] |
| Oral Hypoglycemics | Logistic Regression | 0.58 [0.42, 0.74] | 0.73 [0.64, 0.81] | 0.43 [0.3, 0.56] | 0.49 [0.36, 0.62] | 0.69 [0.62, 0.76] |
|  | Random Forest | 0.72 [0.57, 0.86] | 0.64 [0.54, 0.73] | 0.41 [0.29, 0.54] | **0.53 [0.41, 0.64]** | 0.66 [0.59, 0.74] |
|  | Support Vector Machine | 0.52 [0.36, 0.69] | **0.80 [0.72, 0.88]** | **0.49 [0.33, 0.64]** | 0.51 [0.37, 0.64] | **0.73 [0.65, 0.8]** |
|  | Complement Naive Bayes | **1.00 [1.0, 1.0]** | 0.16 [0.1, 0.24] | 0.30 [0.22, 0.38] | 0.46 [0.36, 0.56] | 0.38 [0.3, 0.46] |
|  | Llama-3.1-8B-Instruct | 0.89 [0.78, 0.98] | 0.29 [0.2, 0.38] | 0.30 [0.22, 0.39] | 0.45 [0.35, 0.56] | 0.44 [0.36, 0.53] |
| Pancreatic Surgery | Logistic Regression | 0.85 [0.82, 0.88] | 0.87 [0.87, 0.88] | 0.28 [0.26, 0.31] | 0.43 [0.4, 0.45] | 0.87 [0.86, 0.88] |
|  | Random Forest | **0.89 [0.86, 0.91]** | 0.84 [0.83, 0.84] | 0.24 [0.22, 0.26] | 0.38 [0.36, 0.41] | 0.84 [0.83, 0.85] |
|  | Support Vector Machine | 0.85 [0.82, 0.88] | **0.88 [0.87, 0.89]** | **0.29 [0.27, 0.32]** | **0.44 [0.41, 0.46]** | **0.88 [0.87, 0.88]** |
|  | Complement Naive Bayes | **0.89 [0.86, 0.91]** | 0.75 [0.74, 0.76] | 0.17 [0.16, 0.19] | 0.29 [0.27, 0.31] | 0.76 [0.75, 0.77] |
|  | Llama-3.1-8B-Instruct | 0.79 [0.76, 0.83] | 0.61 [0.6, 0.62] | 0.11 [0.1, 0.12] | 0.19 [0.17, 0.21] | 0.62 [0.61, 0.63] |

All models have been evaluated on the test subsets. Point estimates and 95% confidence intervals have been calculated by taking the mean, the 2.5^th^ and the 97.5^th^ percentile of the scores that have been calculated over 1000 bootstrap samples. For each dataset, the highest score is printed in bold and in cyan color.

# Supplementary Data


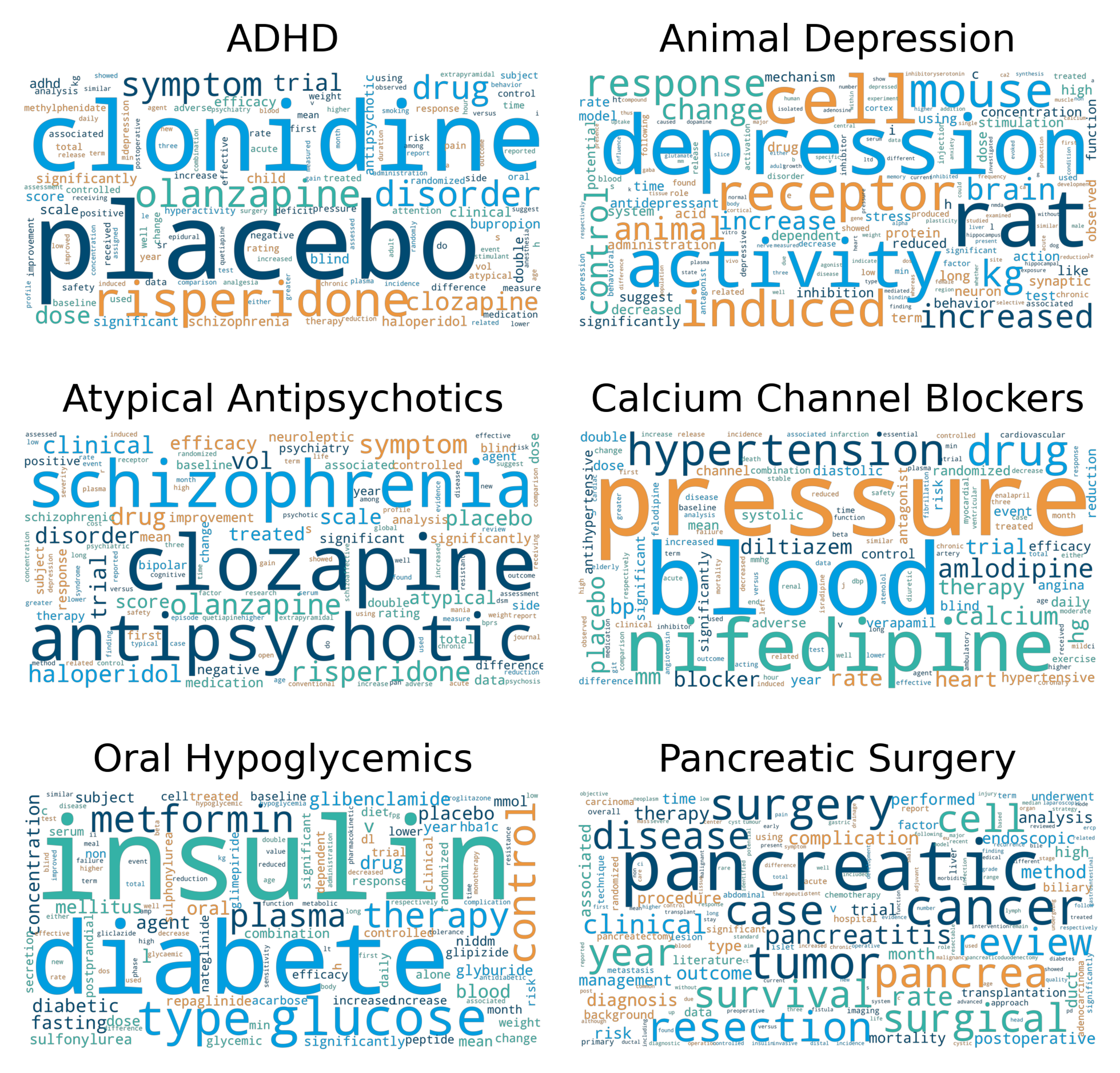


Supplementary Data Fig. 1 | Word Count Analysis More frequently appearing terms are printed larger within the word clouds. After filtering out common stopwords, the most frequent terms are characteristic to the topics of the datasets.


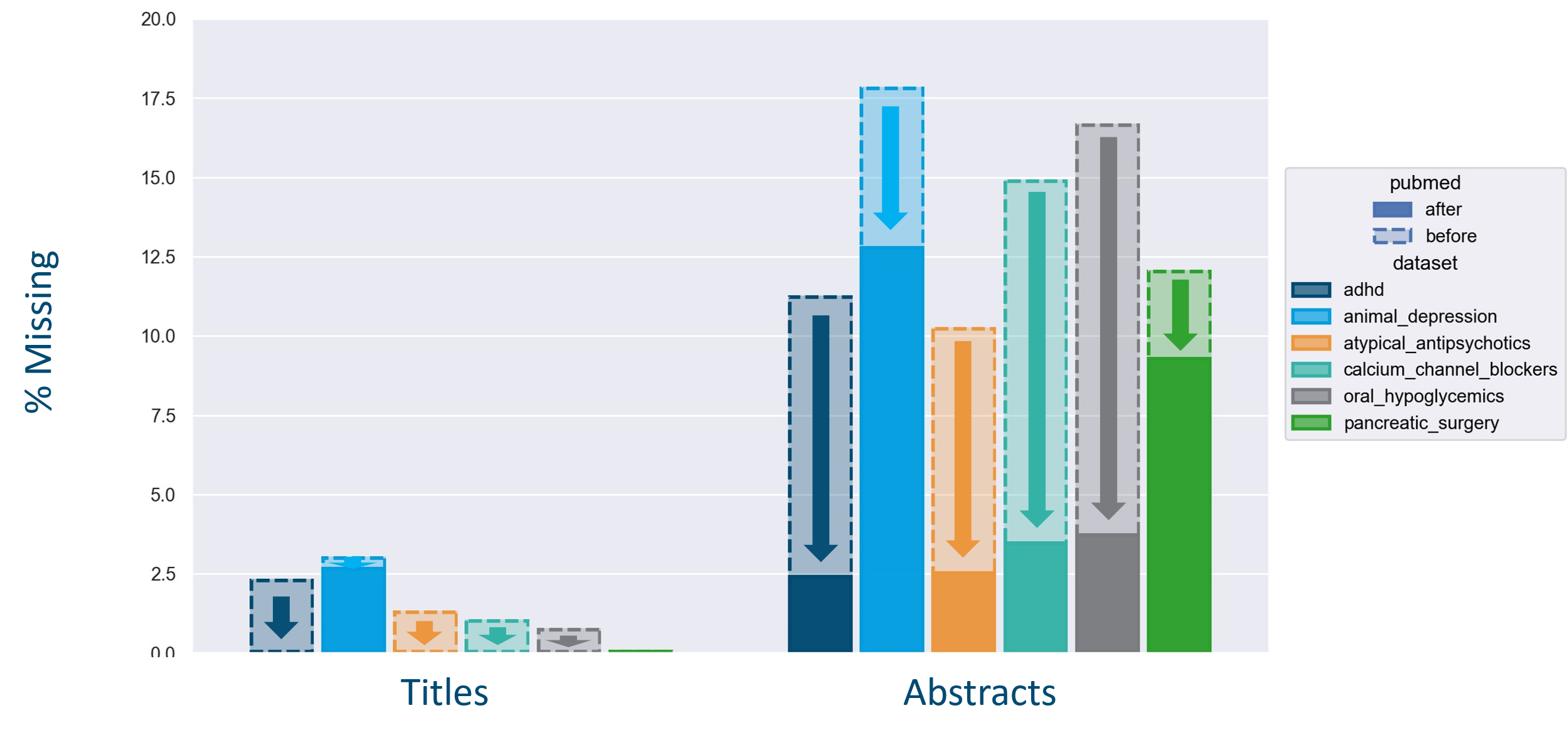


Supplementary Data Fig. 2 | Reduction in articles without titles or abstracts The bars show the relative amount of articles with either missing titles or abstracts before (dashed) and after (solid) calling the PubMed Entrez interface. The number of articles without titles has been largely reduced, and the number of articles without an abstract has been reduced to a varying degree.

Supplementary Data Table 1 | Number of duplicate articles by attribute and dataset. All datasets contain at least two articles with an identical title. The datasets on animal depression and pancreatic surgery further contain articles with duplicate identifiers.

| **Dataset** | **Title** | **DOI** | **PubMed ID** | **OpenAlex ID** | **Web of Science ID** | **Cochrane Central ID** |
| --- | --- | --- | --- | --- | --- | --- |
| ADHD | 1 |  |  |  |  |  |
| Animal Depression | 4 | 2 | 1 | 2 |  |  |
| Atypical Antipsychotics | 4 |  |  |  |  |  |
| Calcium Channel Blockers | 1 |  |  |  |  |  |
| Oral Hypoglycemics | 4 |  |  |  |  |  |
| Pancreatic Surgery | 490 | 12 | 8 |  | 1 |  |

Supplementary Data Table 2 | Inclusion criteria provided to Llama-3.1-8B-Instruct

| **Dataset** | **From Review** | **Selection Criteria** |
| --- | --- | --- |
| Pancreatic Surgery | Probst et al. 2019(74) Probst et al. 2021(50) | **Inclusion:**   - Patients with any kind of pancreatic disease that requires surgery. - All kinds of interventions aimed at affecting the surgical outcome, including medical devices, perioperative management, surgical strategy, drug use, and nutrition. - Systematic cancer therapies, and pancreatic transplantation. - Any other kind of control compared with the above-mentioned intervention including endoscopic retrograde cholangiopancreatography, radiologically guided punctures, or similar interventions. - Outcomes hold mortality, postoperative pancreatic fistula, delayed gastric emptying, post pancreatectomy hemorrhage, bile leak, chyle leak, intra-abdominal fluid collection/abscess, overall morbidity, overall survival, length of hospital stay, and operation time. - Randomized controlled trials. - Systematic reviews with or without meta-analysis which include at least two established literature databases and provide a critical appraisal with validated tools (e.g., Cochrane tool for assessing risk of bias for randomized controlled trials or ROBINS-I for nonrandomized studies).   **Exclusion:**   - Patients with pancreatic diseases that do not require surgery. - Endoscopic retrograde cholangiopancreatography, radiologically guided punctures or similar interventions if not compared to a surgical intervention. - Studies on neo-adjuvant/adjuvant systemic treatment, or pancreatic transplantation. |
| Animal Depression | Bannach-Brown et al. 2016(51) Bannach-Brown et al. 2019(75) | **Inclusion:**   - Any article providing primary data of an animal model of depression or depressive-like phenotype with a suitable control group (cohort of animals that have not been exposed to the method of inducing depressive-like behaviour that was used to create the depressive model). - Animals of all ages, sexes, and species, where depression-like phenotype intended to mimic the human condition have been induced. Including animal models where depressive-like phenotypes are induced in the presence of a comorbidity (e.g., obesity or cancer). - All studies claim to model depression or depressive-like phenotypes in animals. Studies that induce depressive behaviour or model depression and that also test a treatment or intervention (prior or after model induction), with no exclusion criteria based on dosage, timing or frequency. - Studies measuring behavioural, anatomical, and structural, electrophysiological, histological, and/or neurochemical outcomes and where genomic, proteomic or metabolomic outcomes are measured in addition to behavioural, anatomical, electrophysiological, histological, or neurochemical outcomes. - All languages (using automated translations where needed). - Studies must investigate methods or models that induce depressive phenotype/s in vivo, or authors must claim that they investigate a model of depression.   **Exclusion:**   - Review articles, editorials, case reports, letters or comments, conference or seminar abstracts, studies providing primary data, but not proper control group. - Human studies and ex vivo, in vitro or in silico studies. Studies will be excluded, if authors state an intention to induce or investigate only anxiety or anxious behaviour. Studies will be excluded if there is no experimental intervention on the animals (e.g., purely observational studies). - Studies that investigate treatments or interventions, in which no depressive behaviour or model of depression is induced (e.g., toxicity and side-effect studies). - Where metabolic outcome measures are the primary outcome measure of a study. Where genomic, proteomic, metabolic or metabolomic outcomes are the sole outcome measures in a study, they will be excluded. - Studies claiming to induce only anxiety behaviour or a model of anxiety. In cases where both models of anxiety and depression are investigated, the study will be included, and only the depression-related data will be extracted. In the case of data duplication (two or more papers reporting the same data), the paper reporting the smallest dataset or fewest outcomes will be excluded. Studies will be excluded if they model aspects of bipolar disorder, manic symptoms, obsessive compulsive behaviours, panic disorder or psychotic symptoms. |
| Attention Deficit Hyperactivity Disorder | McDonagh et al. 2007(52) | **Inclusion:**   - Populations with pediatric, adolescent, and adult outpatients with either attention deficit disorder or attention deficit hyperactivity disorder - Pharmacologic treatments for attention deficit disorders (e.g., mixed amphetamine salts, atomoxetine, dextroamphetamine sulfate, dexmethylphenidate, lisdexamfetamine dimesylate, methamphetamine hydrochloride, methylphenidate, modafinil). - Outcomes including symptom response, functional capacity, caregiver satisfaction, quality of life, overall adverse effect reports, withdrawals due to adverse effects, or serious adverse effects reported, specific adverse effects (e.g. hepatotoxicity, insomnia, anorexia, effects on growth, abuse potential), misuse/diversion, time to onset of effectiveness, or duration of effectiveness. - Outcomes measured either by the Agency for Healthcare Research and Quality (AHRQ), Technical Review 3 and Mental Measurements Yearbooks. - Controlled clinical trials and good-quality systematic reviews or observational studies with functional or adverse event outcomes. |
| Calcium Channel Blockers | McDonagh et al. 2005(54) | **Inclusion:**   - English language reports of controlled clinical trials in adults with hypertension (blood pressure ≥ 140/90 mm Hg), angina, supraventricular arrhythmia, or supraventricular tachycardia (SVT) and systolic dysfunction (LVEF < 45 %). - Studies with a duration of 2 months or longer. - Interventions including oral dosage forms of nine calcium channel blockers (amlodipine, bepridil, diltiazem, felodipine, isradipine, nicardipine, nifedipine, nisoldipine, verapamil) compared with another calcium channel blocker drug, another oral anihypertensive drug (e.g., ACE inhibitor, beta-blocker, diuretic), or a placebo. - Outcomes for hypertension, angina, supraventricular arrhythmias, and systolic dysfunction included all-cause mortality, cardiovascular (CV) disease mortality, CV events, and quality of life. Additional outcomes included the development of renal failure due to hypertension, symptoms of angina (e.g., episodes of chest pain, use of sublingual nitroglycerin), symptoms (rate or rhythm control) and incidence of stroke due to supraventricular arrhythmias, and symptoms (exercise tolerance, subjective assessments, and New York Heart Association [NYHA] classification) related to systolic dysfunction. - To evaluate effectiveness, include only controlled clinical trials. - To evaluate adverse event rates, include observational studies and clinical trials. |
| Atypical Antipsychotics | McDonagh et al. 2010(53) | **Inclusion:**   - Adults (age 18 years and older and adolescents (age 13 to 17 years) with a DSM III-R or DSM-IV diagnosis of schizophrenia, including other psychotic disorders such as schizophreniform, delusional, and schizoaffective disorders, and including first episode schizophrenia or patients refractory to treatment. - Adults (age 18 years or older) and adolescents (age 13-17 years) and children (under 13 years) with bipolar disorder (manic or depressive phases, rapid cycling, mixed states). - Adults with major depressive disorder. - Older adults (≥ 65 years of age) with behavioral and psychological symptoms of dementia. - Children (under age 13 years) and adolescents (age 13-17 years) with a DSM-III-R or DSM-IV diagnosis for a pervasive developmental disorder, including autistic disorder, Asperger’s disorder, pervasive developmental disorder not otherwise specified (including atypical autism). - Children (under age 13 years) and adolescents (age 13-17 years) with a DSM-III-R or DSM-IV diagnosis of a disruptive behavior disorder, including conduct disorder, oppositional defiant disorder, disruptive behavior disorder not otherwise specified. - Interventions included in this review are aripiprazole, asenapine, clozapine, iloperidone, olanzapine, paliperidone, quetiapine, risperidone, and ziprasidone. - For patients with schizophrenia (including patients with a first episode and treatment resistance), bipolar disorder, major depressive disorder, and behavioral and psychological symptoms of dementia, effectiveness outcomes included in this review are mortality, quality of life, functional capacity, hospitalization, emergency department visits, efficacy as measured by symptom response, adherence (the ability to take medication as prescribed) persistence (the ability to continue taking medication over time), for patients with behavioral and psychological symptoms of dementia, care-giver burden was also included as an outcome of interest. - For children and adolescents with pervasive developmental disorders and disruptive behavior disorders, effectiveness outcomes included in this review are functional capacity, quality of life, hospitalization, emergency department visits, efficacy as measured by symptom response, caregiver burden, adherence (the ability to take medication as prescribed) persistence (the ability to continue taking medication over time). - Outcomes measuring harms across all patient populations are overall adverse effects, withdrawals due to adverse events and time to withdrawal due to adverse events, and specific adverse events. - Randomized controlled effectiveness trials, good quality systematic reviews, and comparative observational studies (cohort studies, including database studies, and case-control studies). - Head-to-head randomized controlled trials, good-quality systematic reviews. If no direct head-to-head evidence exists, placebo-controlled and active-control (conventional antipsychotics) trials were included. - For life-threatening adverse events or those that are important and occur only with longer-term treatment, head-to-head randomized controlled trials, good-quality systematic reviews and meta-analyses, and comparative observational studies (cohort studies, including database studies, and case-control studies) will be included. Before-after studies or single-arm extension studies are included only if follow-up was longer than 2 years. - Randomized controlled trials and comparative observational studies (cohort studies including database studies) examining the relationship between improved adherence or persistence and improved outcomes were analyzed. |
| Oral Hypoglycemics | Chandler et al. 2005^30^ | **Inclusion:**   - Good quality and fair quality studies. - Adults with type 2 diabetes which are of different races, ages, and gender. - Intervention either by sulfonylureas (chlorpropamide, glimepiride, glipizide, glyburide, tolazamide, tolbutamide) or by short-acting secretagogues (repaglinide and nateglinide) - Systematic review or double-blind, randomized controlled trial (including crossover trials) in an outpatient setting (including emergency department). - Clinically relevant outcomes include progression or occurrence of microvascular disease, other complications of diabetes, quality of life and all-cause mortality.   **Exclusion:**   - Paper does not hold original data (e.g. non-systematic review, editorial, letter with no original data). - Studies of multiple oral hypoglycemic drugs (e.g., sulfonylurea/metformin) where the effect of the sulfonylurea can be delineated. - Non-English title and abstract and articles published in abstract form only |

We manually extracted the inclusion criteria for the datasets as we manually extracted them from their underlying systematic reviews. During inference, we loaded the criteria into one string for inclusion and exclusion each, and pasted them into the prompt template in Extended Data Fig. 1.


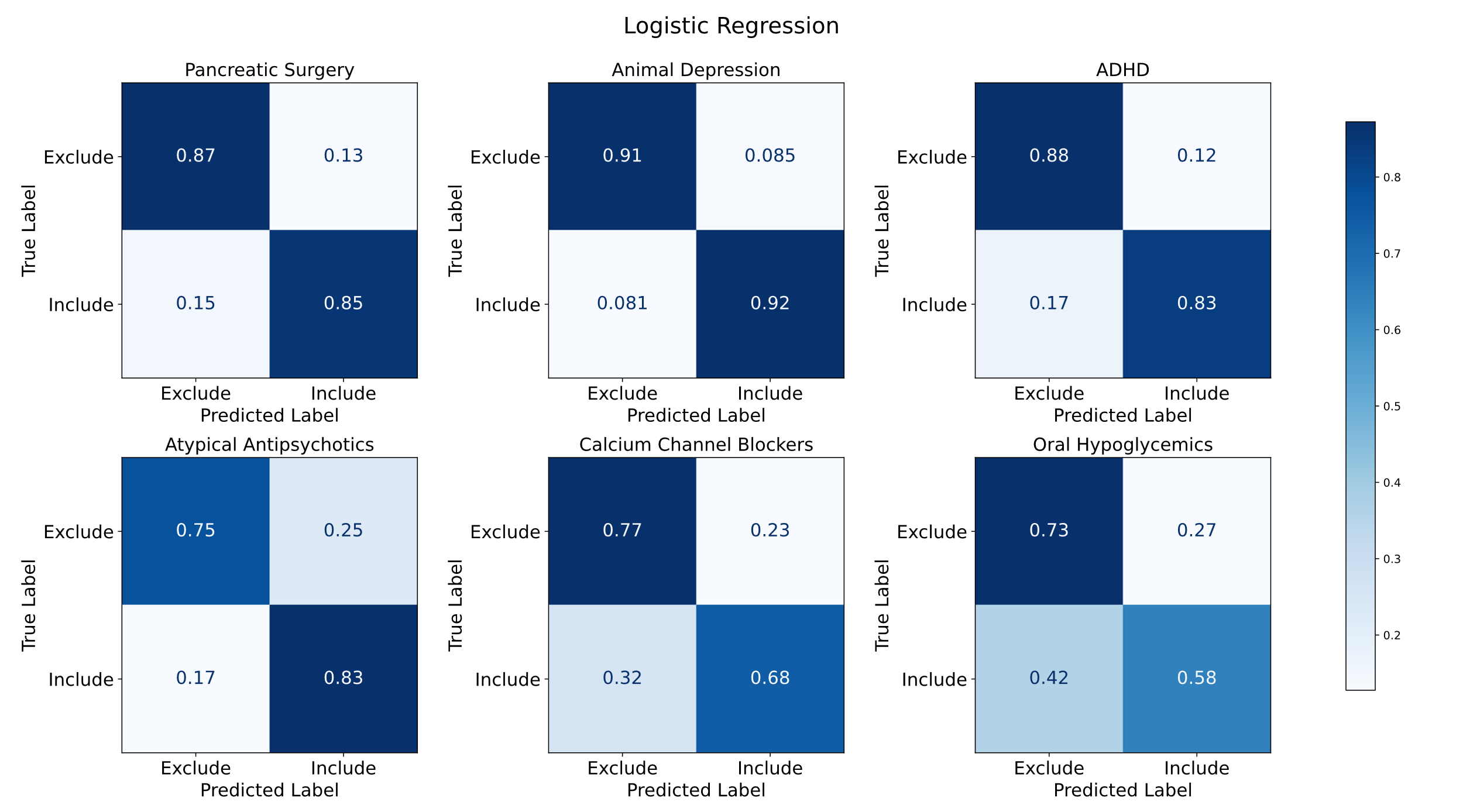


Supplementary Data Fig. 3 | Classification results of the logistic regression classifier


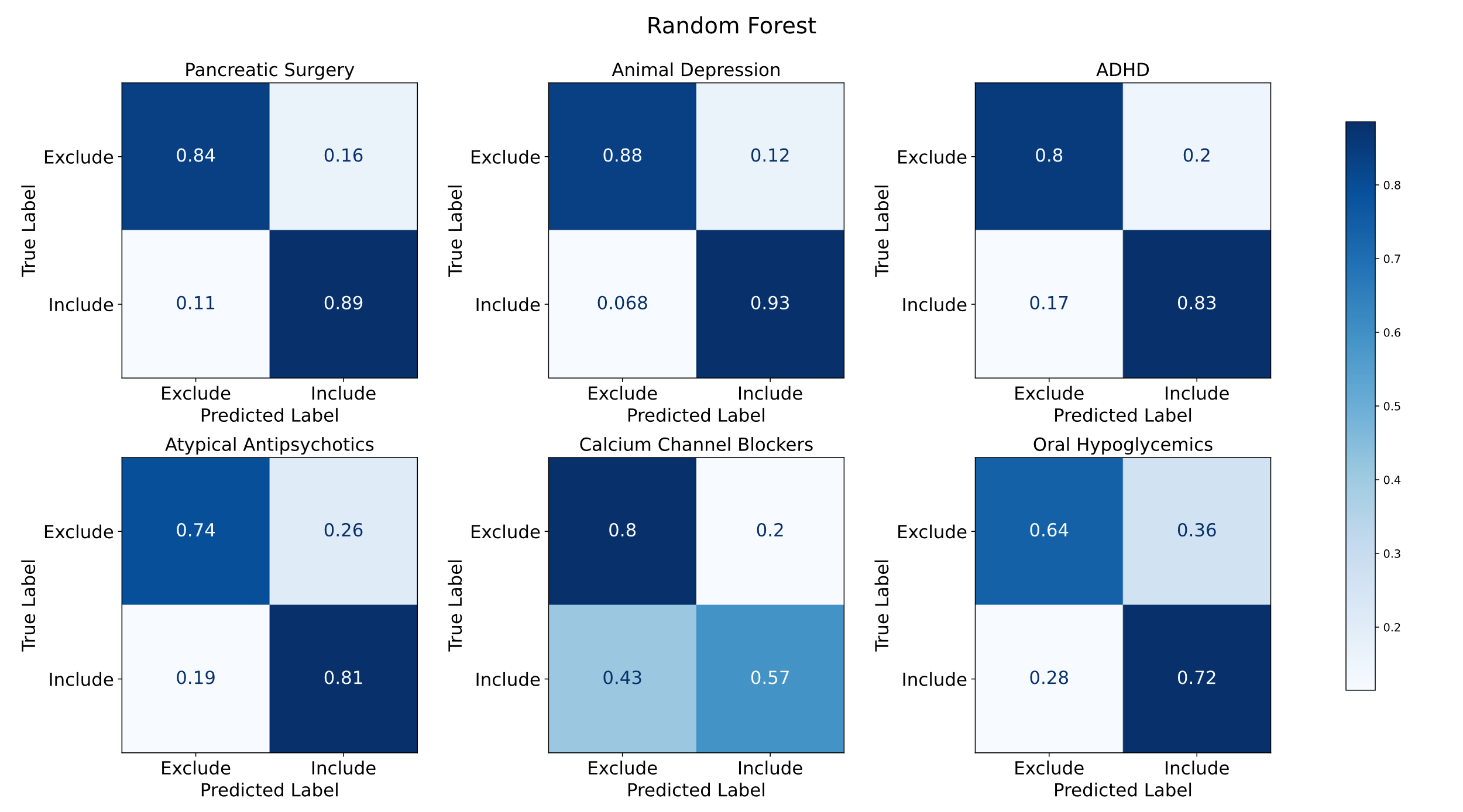


Supplementary Data Fig. 4 | Classification results of the random forest classifier


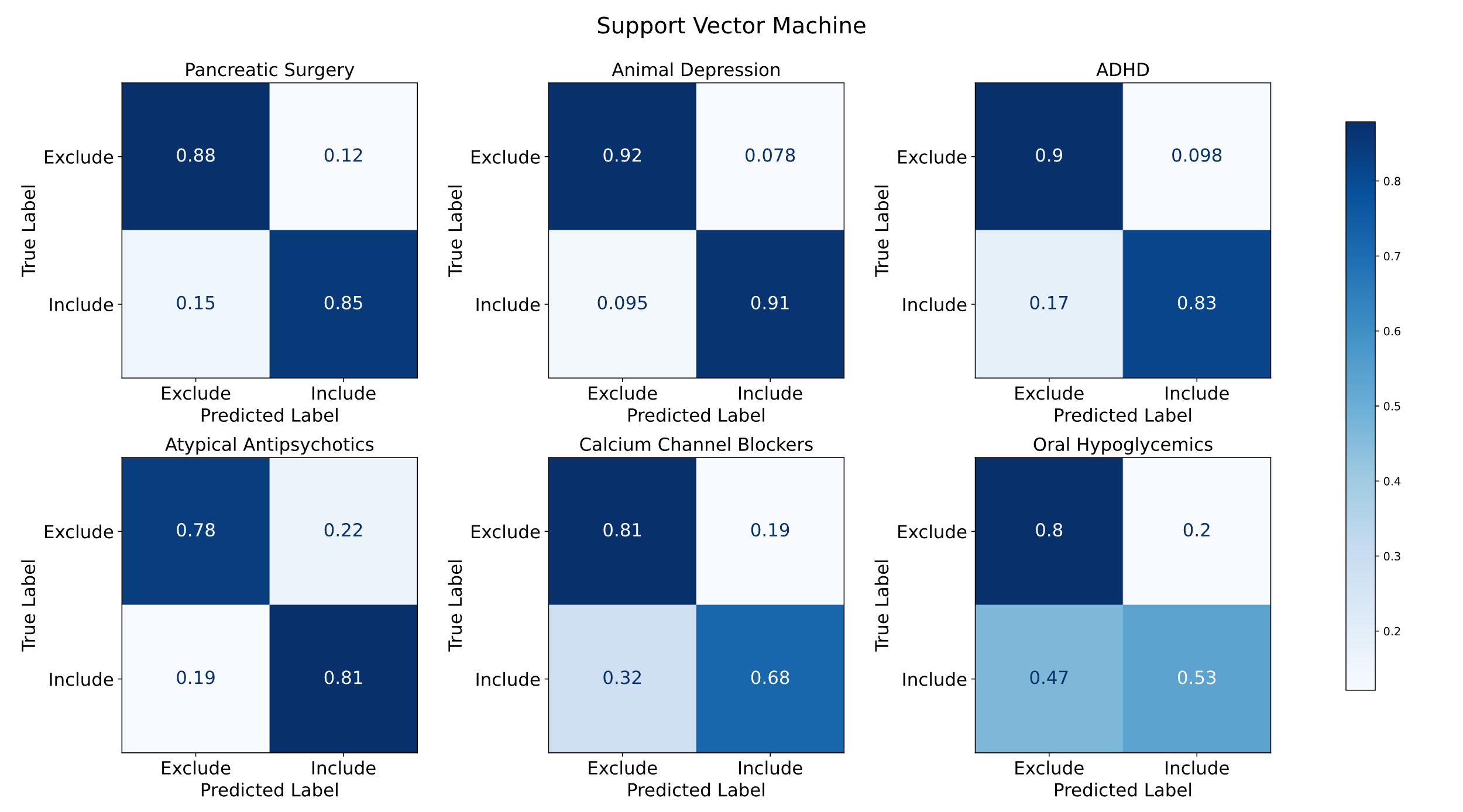


Supplementary Data Fig. 5 | | Classification results of the Support Vector Machine Classifier


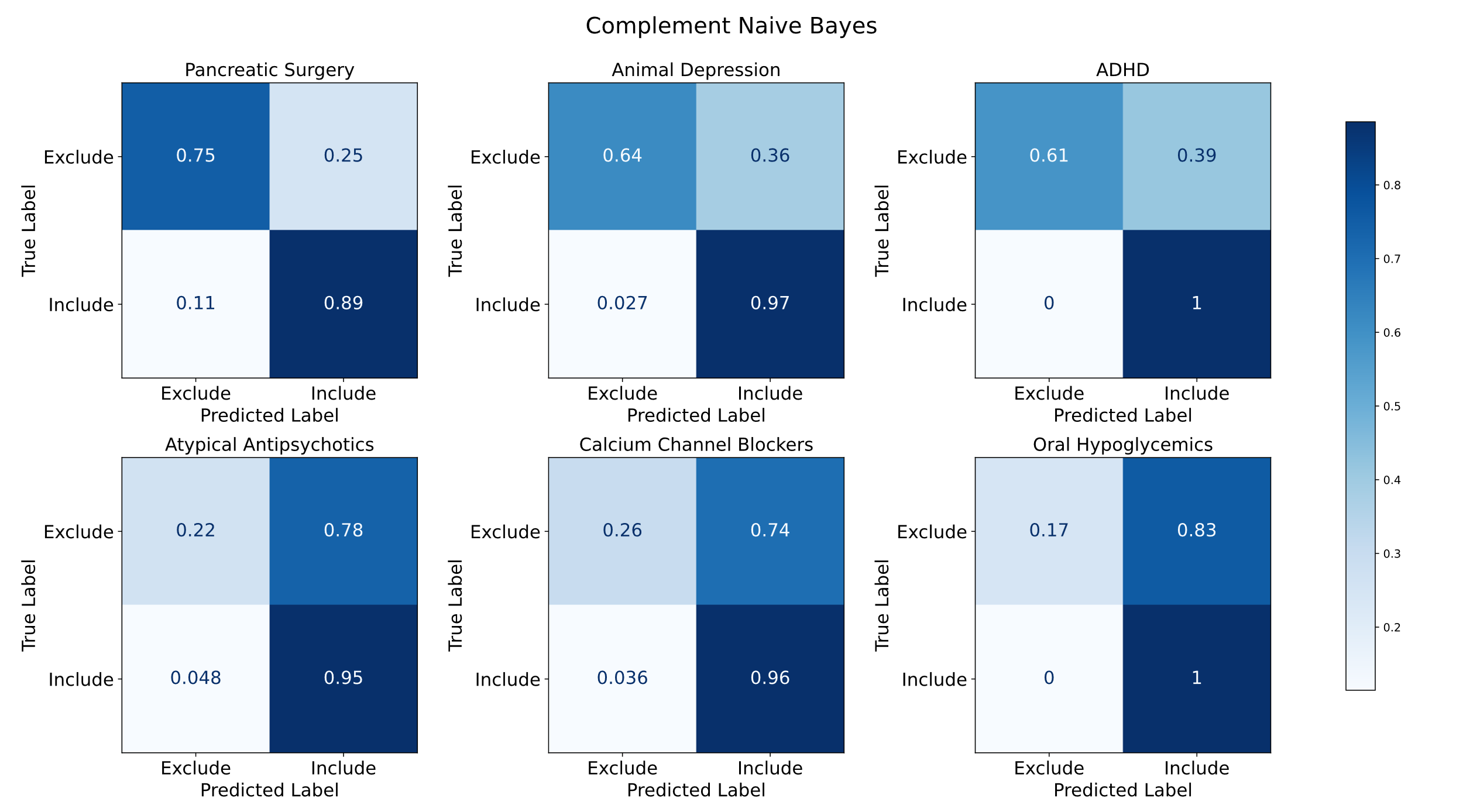


Supplementary Data Fig. 6 | | Classification results of the naïve Bayes classifier


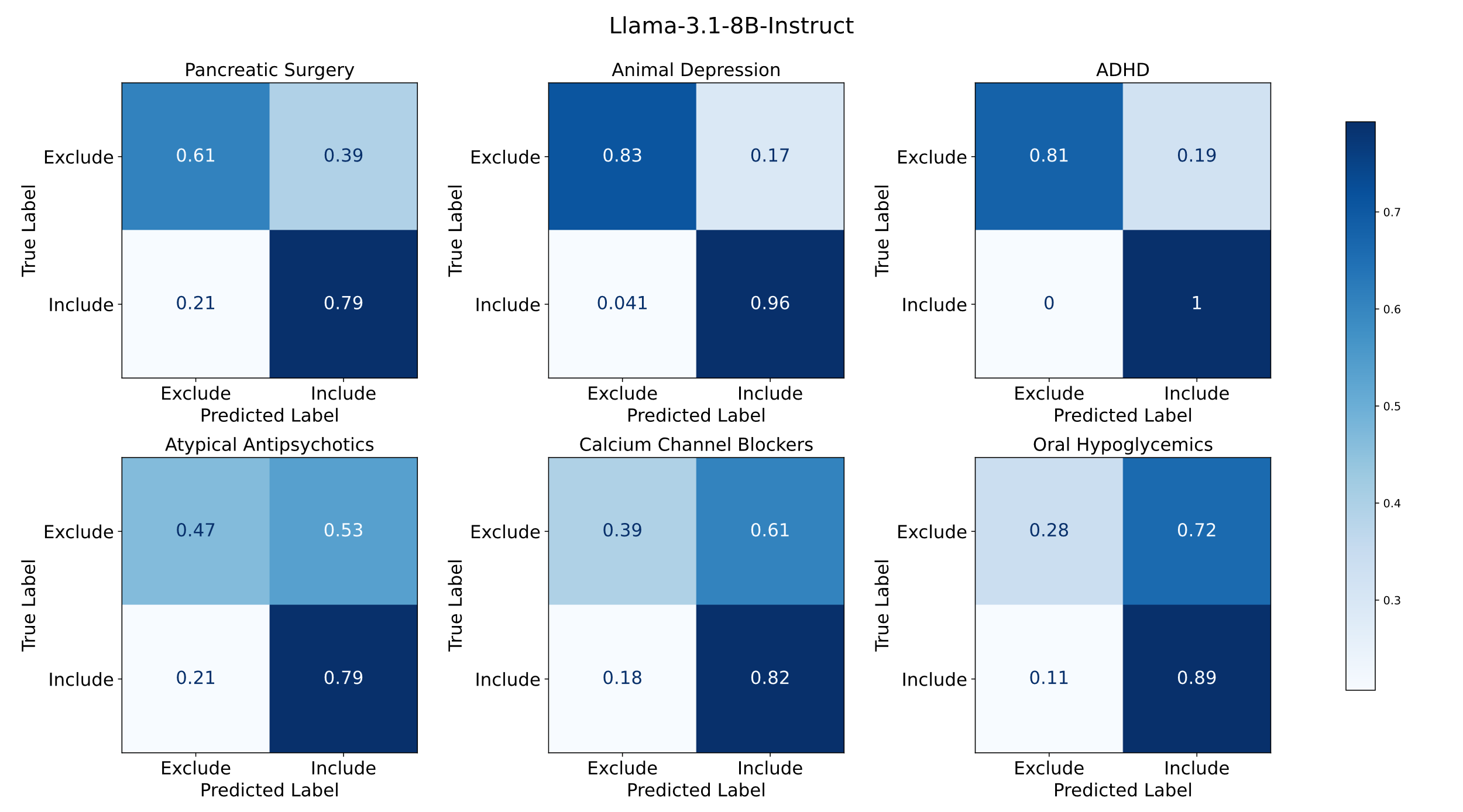


Supplementary Data Fig. 7 | | Classification results of the Llama-3.1-8B-Instruct Model


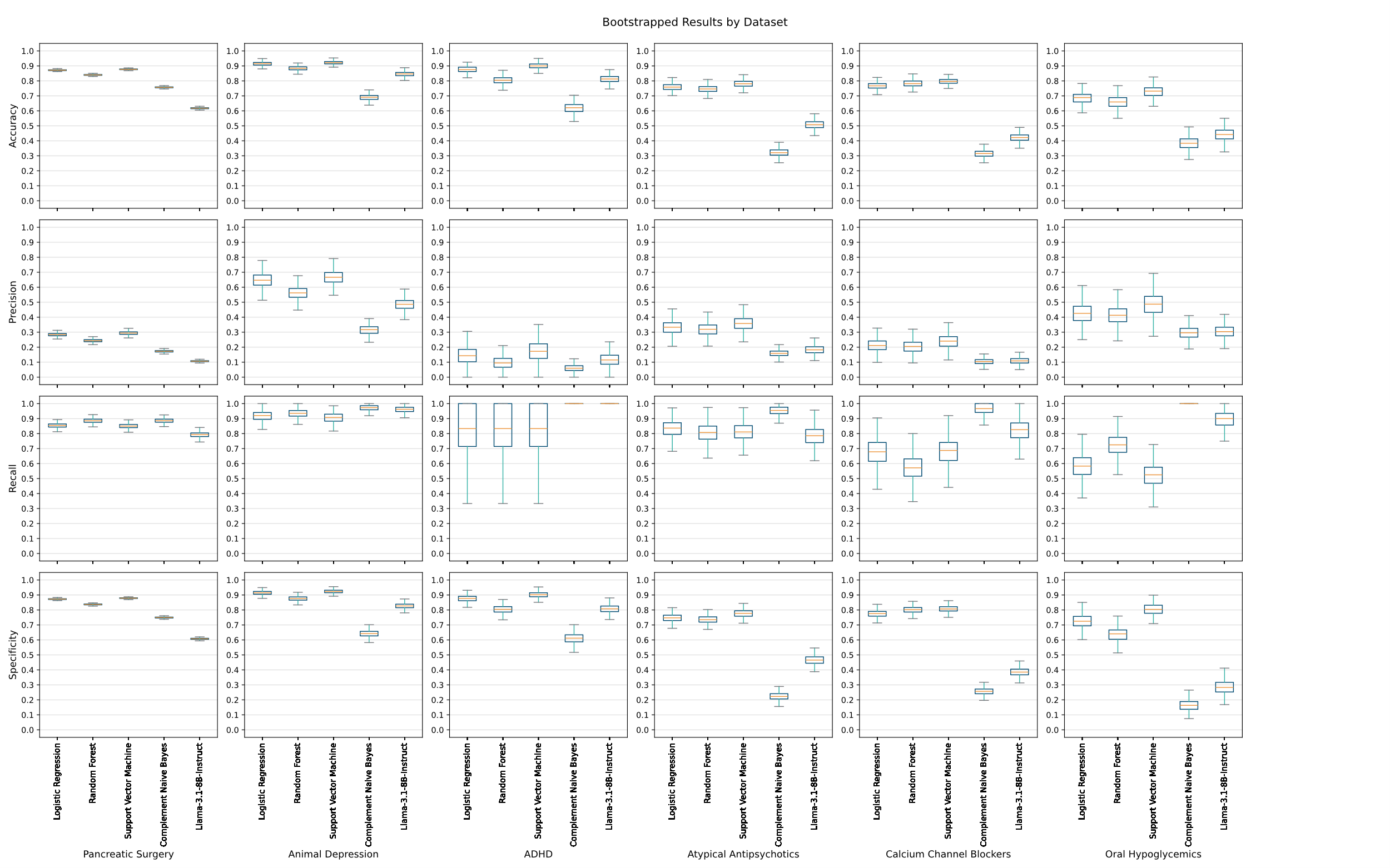


Supplementary Data Fig. 8 | Performance measures for each model grouped by dataset Each subplot represents the combination of one performance metric and dataset. Within each subplot, there is one boxplot per model, showing the score, it has achieved.


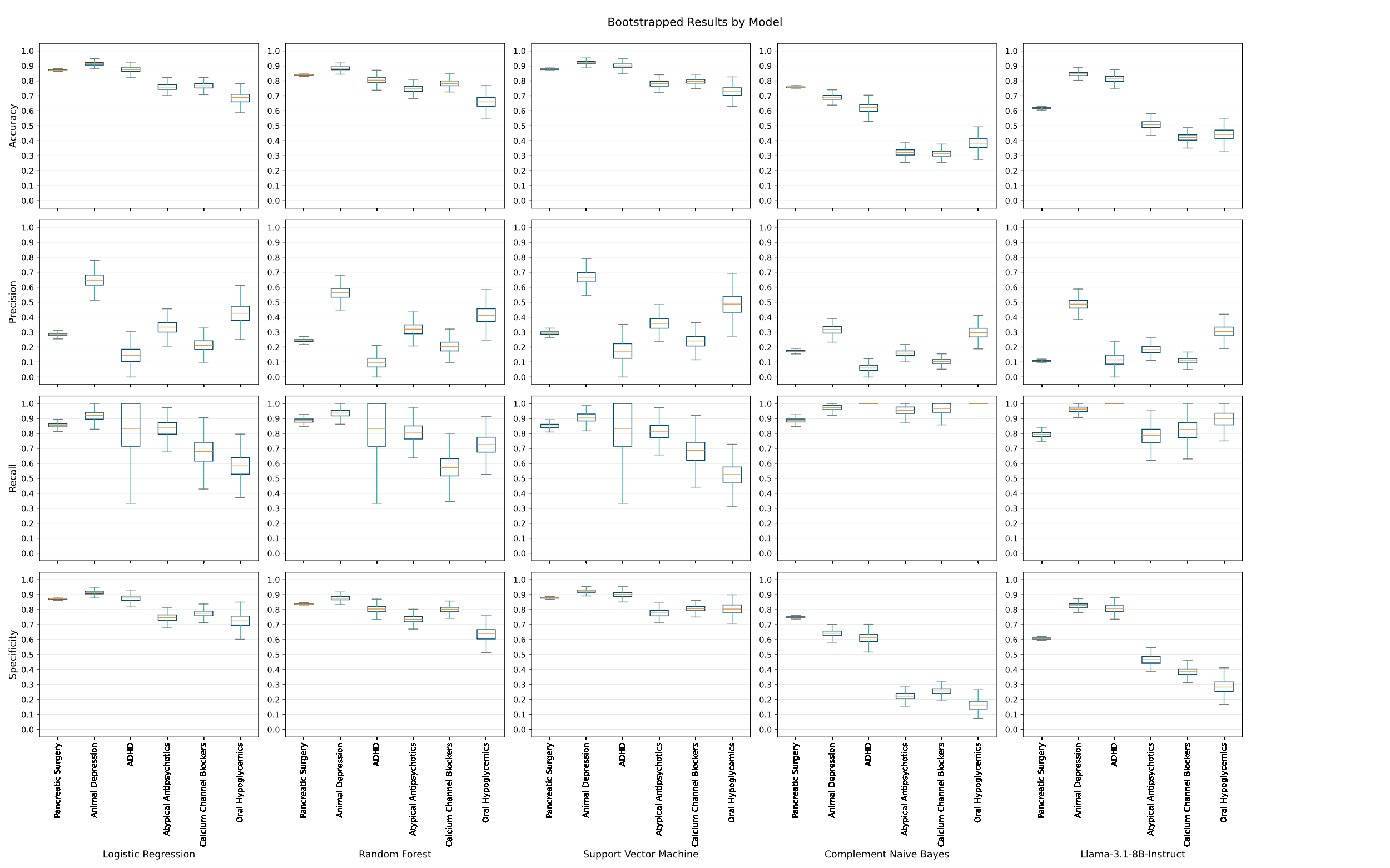


Supplementary Data Fig. 9 | Performance measures for each dataset grouped by model Each subplot represents the combination of one performance metric and model. Within each subplot, there is one boxplot per dataset, showing the score, it has achieved.
